# Measurement of the Geometric Center of a Turnout for the Safety of Railway Infrastructure Using MMS and Total Station

**DOI:** 10.3390/s20164467

**Published:** 2020-08-10

**Authors:** Arkadiusz Kampczyk

**Affiliations:** Department of Engineering Surveying and Civil Engineering, Faculty of Mining Surveying and Environmental Engineering, AGH University of Science and Technology, al. A. Mickiewicza 30, 30-059 Krakow, Poland; kampczyk@agh.edu.pl

**Keywords:** double slip turnout, outside slip, turnout deformation, curve versine, turnout measurement, magnetic-measuring square, MMS, curvature in the turnouts, linear measurement procedure, safety in railway infrastructure

## Abstract

The turnouts in railway infrastructure constitute bottlenecks, limiting the capacity of the entire railway network. These turnouts force speed limits due to their design and geometry. The need to ensure the proper technical condition of turnouts has prompted ongoing scientific research and the use of modern technological solutions. Until now, there have been no tests for the correct location of the geometric center of a double and outside slip turnout with the related geometric relationships. Therefore, the main objective of this research was to demonstrate the position of the geometric center of a double slip turnout and the geometric conditions of the curves of circular diverted tracks by measuring the horizontal versines and geometric irregularities of turnouts. The application of this surveying method, with reference to obtuse crossings and arising from geometric dependencies in the double and outside slip turnout, is defined and implemented (also known as a method for checking the correct location of the geometric center of a turnout—Surveying and Monitoring of the Geometric Center of a Double and Outside Slip Turnout (SMDOST)) via the Magnetic-Measuring Square (MMS) and electronic Total Station. This method also recommends measuring the horizontal versines of the diverted tracks. This paper presents the results of field measurements while using the SMDOST and MMS methods, which were applied to carry out an analysis and evaluation of the turnout geometry conditions, thereby presenting the irregularities that cause turnout deformations. The validity of the SMDOST method using MMS and Total Station was thus confirmed. The observations from the conducted research indicate that neglecting measurements of the geometry of the turnouts resulted in additional irregularities in their conditions.

## 1. Introduction

The surveying of railway turnouts (railway switches) is one of the main survey tasks needed to ensure safety. The results of monitoring turnout geometry are essential for planning and optimizing maintenance work. The railway network is a system of interconnected railway roads managed by the infrastructure manager. The railway track, including turnouts and diamond crossings, is an element of the railway infrastructure [1]. The most critical element of a properly functioning infrastructure is primarily the technical condition of the railway’s surface. Turnouts are an extremely important element of railways and have a direct impact on limiting the speed of the rolling stock due to their functions, structural and geometric complexity, and cooperation with the rolling stock. Railway lines are subject to requirements under both European and national law, and railway interoperability should always ensure safe and uninterrupted train movement [2]. The turnouts and diamond crossings on railway lines and marshalling yards constitute bottlenecks that limit the capacity of the entire railway network. These turnouts force speed limits due to their design and geometry, which, in turn, affect capacity constraints. Surveying the state of changes in turnout geometry is one of the most important scientific issues.

The increase in demand for high-speed rail systems in the Member Countries of the European Union and the high level of investments in such systems have become a technical and organizational challenge for the manufacturers of all relevant components and for operators and infrastructure managers. Operators are facing ever-increasing demands to provide improved driving comfort, increased reliability, and ensure the durability of all components of the railway track (i.e., the permanent way and the railway surface) [3]. The new design solutions for double slip turnout on strine–concrete switch sleepers should allow the operating speed to be increased to 120 km/h on the main track and *V* ≤ 40 km/h on the deviated track (diverted track) [3].

Many geopolitical, economic, and social changes taking place in the modern world have generated an increase in citizens’ expectations for fast, safe, and reliable transport. The answer to these expectations is the development of high-speed rail systems, which entails innovation in the railway industry. Solutions implemented on high-speed rail system networks are gradually migrating to conventional rail, urban railway, and metro systems, thereby increasing the competitiveness of the entire sector [4]. According to Márquez et al. [5], the European Commission estimates that passenger traffic will double and that freight traffic will triple by 2020. In the same period, a reduction of 30% in the lifecycle costs (LCC) of track assets is desirable. The remote condition monitoring (RCM) of track-side equipment such as turnouts and level crossings can help increase reliability, availability, maintainability, and safety (RAMS). Nevertheless, the application of the LCC model to RCM has thus far been neglected. Márquez et al. [5] took up the subject of LCC for RCM using a real case study, showing how the costs and benefits of RCM can be assessed. Railway turnouts work under difficult environmental conditions, but their reliability requirements are high due to safety and economic factors. Once implemented, their maintenance depends on the data collected regarding their condition and decisions on appropriate corrective actions. The more regular the data collection and decision cycle are, the greater trust the operator will have in an efficient and correct service [6]. Proactive maintenance entails carrying out maintenance activities that are based on operating hours or the number of hours of operation instead of planning maintenance only when really needed. This requires an effective combination of data collection, analysis, presentation, and decision-making processes [6].

The problem of the durability of turnouts and rails remains open to technological research in order to determine the relevant structural relationships and mechanical characteristics [7,8]. The most complex and important construction elements of railroads are turnouts, which are particularly exposed to abrasive wear, fatigue, and shape changes as a result of the high dynamic cyclic loads that occur during the passage of railway vehicles [7]. Railway turnouts are one of the most critical elements of railway infrastructure [9,10,11]. The complex physical phenomena within the switch, which are associated with significant forces between the vehicle in motion and the track, require special attention to be paid to turnouts. Increasing turnout speeds, both the main track and on the deviated track, require increased turnout geometry and blade (switch rail) length [9]. Highly reliable and simple turnout maintenance is required due to the expected high availability of the line, which makes it necessary to newly explore turnout technology. Modern turnout technology is characterized by constant development that is driven by the growing expectations of leading infrastructure managers regarding high reliability and low maintenance costs [9].

Khouy et al. [12] argued that each year, a significant part of the budget should be devoted to the inspection, maintenance, and renewal of turnouts. The application of a cost-effective maintenance strategy can help achieve the best performance at the lowest possible cost. In Sweden, the geometry of turnouts is checked at specific intervals with the STRIX/IMV 100 measuring wagon (track geometry car). Xu et al. [13] stated that turnout wear is a common mechanism that destroys turnouts. Other studies also demonstrated the influence of the wear of turnout sections on the behavior at the wheel–rail contact points and their dynamic actions. The wear of profiles interferes with the distribution of the wheel–rail contact points, changes their positions along the longitudinal direction, and affects the dynamic impact of the vehicle and the turnout [13].

The dynamic passage of rolling stock through turnouts is reflected by their condition. The experimental and numerical studies carried out indicate that the axle load of the train [14], the running directions of the rolling stock, the dynamic loads due to the influence of the wheel profile of the rolling stock [15], the quality of the rolling stock passing, the total process of the wheel passing through the turnout [16], the type of crossing (frog) and the sort and type of turnout, the wheel–rail contact interaction, damage to the crossing and turnouts (material, number, etc.) [17], the wheel profile-crossing elements [17,18,19], the turnout switch sleeper stiffness, the ballast condition, and the elastic properties of turnout support structures [20,21] have an impact on the efficiency, effectiveness, and performance of the turnout and its components. Turnouts and diamond crossings are key elements in railway networks. Any failure usually leads to delays and cancellations of trains, which has a negative impact on the quality of service provided, railway safety, and operating costs. Turnouts are multi-component systems, and, to actively prevent failures with undesirable consequences, it is necessary to successfully anticipate problems at a component level [22].

The current regulations inform the measurements of the actual geometry of exploited turnouts and diamond crossings, as well as their visual inspections, technical testing, and maintenance [23,24,25,26,27]. EN 13232 (nine parts of the standards) determines the design and production of turnouts and diamond crossings [28]. These regulations support the monitoring of railway infrastructure components for interoperability in order to ensure the safe and uninterrupted passage of trains, thereby meeting community requirements for rolling stock on the rail network. The turnout is a special multi-track structure made of rails, steel sections, and other elements, which allow railway vehicles to pass from one track to another at a certain speed [23]. The main types of turnouts are as follows:

- Single point (single turnout, point);
- Slip turnout;
- Curved slip (Y-points);
- Three-way turnout.

**Figure 1:**
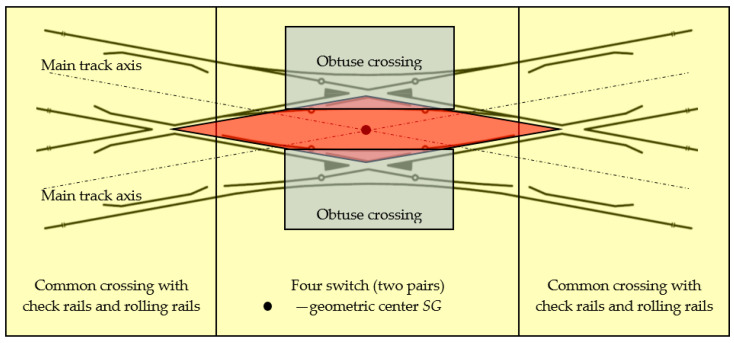
A diagram showing a double slip turnout—Rkpd_iwew_, where the red color indicates the quadrilateral of the turnout.

A double slip turnout (Rkpd_iwew_) is composed of the following (Figure 1):

- Four switches (two pairs);
- Two obtuse crossings; and,
- Two common crossings (single crossing) with check rails and rolling rails.

Depending on the location of the switches, some turnouts feature blades inside the turnout quadrilateral—a double slip turnout (known as “English”)—while some turnouts feature blades outside the turnout quadrilateral—an outside slip turnout (known as the “Bäseler system” or “shortened English”) [29]. Under the term, a turnout quadrilateral is the area that is contained from the normal to the bisectoral angles of common crossings (at English turnouts), obtuse or triple (at Bäseler system turnouts), carried out by their mathematical points [29]. The double slip turnout—English—is created by building two connections in a diamond crossing in diverted (deviated) directions. This turnout is composed of two common crossings (single crossing), two obtuse crossings, four pairs of switches, and connecting rails (Figure 1).

This turnout provides a passage in four directions: two straight directions and two turns deviated (diverted). When the blades are placed inside the quadrilateral of a turnout, there is a limitation in the length of the tangents to the curves of the diverted tracks, which prevents the construction of turnouts with a diverted track radius greater than 265 m. Limiting the value of the turnout angle entails that, for the largest turnout used on the PKP (Polish State Railways S.A., in Polish: Polskie Koleje Państwowe S.A., PKP), the radius of the diverted track will be, as follows [29]:

- 205 m at a tangent of crossing angle of 1:9 and an S42 turnout type; and,
- 190 m at a tangent of crossing angle of 1:9 and 49E1 and 60E1 turnout types.

The diamond crossing at a tangent of crossing angle of 1:9 is a skeleton of single and double slip turnouts, with the blades being located within the boundaries of the quadrilateral turnout and with the blades located outside the boundaries of the quadrilateral turnout [30]. A distinction is double slip turnouts with points of intersection whose blades are within the limits of the quadrilateral turnouts (for smaller radius), and with points of intersection whose blades are outside the limits of the quadrilateral turnouts (for larger radius) [31]. The maintenance of the railway track is characterized by a variety of existing turnouts, diamond crossings, and adjustment switches (expansion joints, breather switches, and special expansion switches) with different shapes of curves and inclinations, as well as different shapes of switch sleepers. Regular assessments of all turnouts, diamond crossings, and adjustment switches should be done according to the speed and ballast, as required [32].

The outside slip turnout—the Bäseler system—is created by building in a diamond crossing two connections in the diverted direction, in such a way that the outer courses of these connections intersect one course of each of the two intersecting tracks. This turnout consists of two obtuse crossings, two triple crossings, four switches, and connecting rails. The characteristic structural elements of these turnouts are double and triple crossings. The arrangement of blades in the outside slip turnout outside the quadrilateral of the turnouts contributes to the lack of a limit on the radius of the diverted track, which is typical for double slip (English) turnouts. This makes it possible to build turnouts with a tangent of crossing angle of 1:9 and diverted track radii of 300 and 500 m, as well as turnouts with a tangent of crossing angle 1:6.6 and a 190 m radius [29].

The surveying and monitoring of the geometric location of the turnout’s center point is one of the most important scientific issues and it is reflected in the linear rail transport infrastructure facilities in operation. A thorough review of the scientific and technical literature shows a lack of publications in this area. Therefore, the present study offers a detailed description and continuation of previous research work [2,33,34,35,36,37,38,39]. In [33], characteristic points for the measurement of turnouts were defined, including double slip turnout. In [38], a digital track gauge that was designed to measure the characteristic geometric dimensions of tracks, turnouts, and diamond crossings, especially within the crossing, was presented. In turn, the work in [34,35,36,39] presented the results of studies on the measurement of versines (exploitation, assembly) on the diverted track of a single point turnout via the innovative Magnetic-Measuring Square (MMS) method [39].

This paper contains the results of research on the actual state of Rkpd_iwew_ geometry, which is a defined and implemented surveying method with reference to obtuse crossings and it arises from the geometric dependencies in double and outside slip turnout (also known as a method for assessing the correct location of the geometric center of a turnout, Surveying and Monitoring of the Geometric center of a Double and Outside Slip Turnout—SMDOST) using MMS and the electronic total station TC407 Leica. The main objective of this research was to demonstrate the following:

- The position of the point condition geometric center SG of the double slip turnout.
- The geometric condition of the curves of circular diverted tracks by measuring the horizontal versines.
- The geometric irregularity condition of turnouts.

The measurements were covered by an exploited double crossover turnout with spires inside a quadrilateral Rkpd_iwew_ No. 9 type S49/1:9/190, which was constructed from S49-type rails with a tangent of crossing angle of 1:9 and a radius of 190 m. This was built on the main additional track, No. 6, and assigned to Railway Line No. 131 Chorzów Batory–Tczew in the territory of the Silesian Voivodship (in Polish: Śląskie), Lubliniecki County, Poland.

For the state of the geometric irregularities of double slip turnouts with blades inside the Rkpd_iwew_ quadrilateral, the research was additionally confirmed by monitoring the four turnouts within the S49/1:9/190-type double obtuse crossing (No. 134, 136, 182, and 139) in the territory of the Silesian Voivodship, Tarnogórski County, Poland.

The exploitation speed on the main and diverted tracks of the turnouts covered by the tests was *V* = 40 km/h, whose acceptable deviations (for track gauge and cant (superelevation parameters)) have already been established.

The SMDOST was also used for double cross-junction with blades outside the quadrilateral Rkpd_izew_. Controversially, in the relevant legal regulations [23], the slip turnouts (double and outside slip turnout and single slip turnout) do not require their versines to be measured. The values of the exploitation versines reflect the conditions of the curves of the diverted track. It is also surprising that the geometric positions of the double and outside slip turnout (Rkpd) center have, to date, only been determined by the intersecting axes on the main track of the turnout. This was confirmed by the proprietary SMDOST study results.

This research shows the practicality and usefulness of using the SMDOST method with MMS, especially in places that are difficult to access (e.g., obtuse crossings and check rail turnouts). Elements affecting the anomaly of Rkpd’s geometric center position and its deformation are shown. The durability of turnouts depends not only on the material properties and turnout design, but also on the operating conditions and turnout geometry. The presented method of surveying, with reference to the obtuse crossing and the resulting geometric dependencies in the double and outside slip turnout, pays special attention to the state of the geometric dependencies of the monitored turnout. This method offers in-depth insights and conclusions from the turnout operation process. The new insights that are contained in this article can be used not only as a reference point for the inspection and maintenance of turnouts but also for their installation. This method can be used for the robust planning, decision making, analysis, and condition assessment of the geometry of a turnout’s double and outside slip. This paper this makes a profound contribution to the interdisciplinary fields of surveying, traffic construction, and traffic engineering. The conducted research is an element of the surveying and diagnostic work in the discipline of civil engineering and rail transport.

## 2. Related Work

Turnouts are an essential element of any railway network. However, they are also capacity constraints [40,41]. There are many types of turnouts, and their properties vary depending on their destination (use). The main feature of a turnout is its projected speed. In practice, each turnout is influenced by adjustments to the layout of a given location. A compromise between space, cost, railway-line speed, and efficiency in the choice and location of turnouts should be considered. Bemment et al. [40] presented the constraints of turnouts in the performance of a railway network, demonstrating that these constraints arise because turnout (track connection) designs have evolved over time in order to meet a specific objective, which, at the same time, means that turnouts may not be optimized to provide the performance that a modern network requires. The authors defined the formal requirements for track connection solutions, arguing that traditional solutions do not meet all of the requirements, and additionally presented an innovative solution for track connections called the “Repoint” solution.

The issue of railway performance and capacity is highly dependent on the state of the infrastructure. Capacity varies depending on factors, such as train speed, the location of stops, train heterogeneity, the distance between train signals, and timetable status. Capacity is an important problem in the railway industry, and the efficient use of infrastructure is a difficult requirement for railway undertakings [42,43,44]. Rail track connections provide the necessary flexibility for the railway network. However, turnouts cause single points of failure and limit capacity [45]. Turnout failures in high-traffic passenger rail systems cause disproportionately long delays. It is recognized that, as equipment failures are eliminated, reliability and availability become contributory factors to a lack of reliability [46].

Turnouts are an expensive and critical point of feature of the railway system, as they are exposed to adverse operating loads as compared to a simple railway track and, therefore, require regular maintenance [11]. Sae Siew et al. [11] noted the torsional effects of railway turnout crossings on track-support structures. Turnouts, by virtue of their construction and purpose, are one of the subassemblies that form part of the railway infrastructure with the greatest number of accidents featuring railway vehicles [47]. The main points of derailments during train passage are the points of the switch and crossing (crossing). When the wheels of the rolling stock pass through a switch on a crossing, forces can be twice as high as when driving on the track. Kisilowski and Kowalik [47] presented the results of research on the application of a visual system for monitoring the diagnosis of individual elements of railway turnouts. This diagnostic system performs a real-time check of the surface condition of the switch point and crossing and determines the geometric values of both elements.

Hamarat et al. [48] noted that a turnout is the basic infrastructure for managing the flexibility of rail traffic. The turnout imposes restrictions on the operation of trains, which results in dynamic reactions to the high amplitude of interactions between trains and turnouts. A numerical model that was capable of determining the impact forces was thus developed to evaluate the dynamic behavior of turnouts and their influence on turnout elements such as the supports and ballasts [48].

Hamadache et al. [49] emphasized that the railway turnouts and level crossings (railway switch and crossing—S&C) have a very complex structure. The complexity of railway switches and crossing systems makes them vulnerable to breakdowns and malfunctions that can ultimately cause delays or even fatal accidents. Thus, we must develop suitable monitoring techniques to handle fault detection and diagnosis (FDD) in railway S&C systems.

In most countries, railways are a main part of the transport infrastructure. Thus it is essential to apply good maintenance strategies for railway networks to avoid service disruptions and ensure system security. For example, the conditions of the railway track must be regularly monitored to detect faults at an early stage before they become serious problems [50]. Mańka [51] focused on the need for risk analysis and process approaches in safety management, including a safety management system (SMS) and maintenance management system (MMS), featuring additional systems as compared to typical railway systems, such as control command and signaling, the transport process, and the maintenance and repair process. In [50], the use of train running measurements for monitoring railway tracks was examined. An Irish Rail train in operation was instrumented with accelerometers and a Global Positioning System (GPS).

Salvador et al. [52] noted that the maintenance of railway tracks is becoming a real challenge for railway engineers due to the need to meet increasingly higher quality requirements while using cost-effective procedures. Gomon and Gómez [53] referred to cross-border rail transport as a special case of rail transport that creates many difficulties in crossing state borders. These difficulties are due to factors including other power systems, other control systems, different traffic management regulations, and even communication problems due to different languages. The authors also noted that railway workers often rely on their experience.

The geometric arrangements of the track connections are characterized by the following features [8,54]:

- Having a decisive influence on the operational parameters of railway lines;
- Having a significant impact on the construction costs of new lines; and,
- Being marked by a low susceptibility to change.

One of the basic tasks of detecting threats on a railway track is to monitor the increases in the track’s degradation from a state of full operational suitability, through a state of deteriorating performance, to a state of endangering traffic safety [55].

Kwiatkowska [56] described the process of diagnostic work on turnouts using innovative research methods, focusing on the application of a Scorpion three-dimensional (3D) scanner for the 3D profile measurements of turnout components for the early diagnosis of damage to the blade, stock rail, and crossings. The results were also presented based on testing the dynamic interactions generated by rolling stock movement; the authors noted that the purpose of conducting diagnostic and dynamic tests is detecting damage to turnout components.

Minbashi et al. [57,58] presented the results of studies on the assessment of the geometric quality of railway turnouts using power spectral density (PSD). The results indicate that PSD in the form of a continuous curve may show irregularities in the wavelengths and amplitudes of their turnouts. The measurement of changes in the vertical geometry at turnouts is exposed to different types of work; as a result, a method for measuring the vertical position of track geometry under non-operational conditions (without stress) was introduced in order to show track degradation [10]. The state of behavior of turnout geometry in the vertical plane was shown, depending on whether the turnouts were located in the main or diverted track [10].

One developmental method for monitoring turnouts involves an inertial measurement using the Electronic Analysis System of Crossing–Portable (ESAH-M) system. Sysyn et al. [59] noted, however, that the accuracy and sensitivity of such measurements are affected by many factors, which may lead to high measurement uncertainty. The inaccuracy of measurements and data processing depend largely on the relationship between the point of impact at the nose of the crossing and the position of the sensor [59]. The monitoring of turnout elements was carried out using the ESAH-M system, based on a dynamic measurement and a displacement system using a remote video meter. In turnout components (e.g., in the crossing), high contact forces are exerted at the wheel–rail contact point through the wheel’s impacts on the wing rail and at the nose of the crossing. This results in significant wear and tear [60]. Moreover, there was no ballast, no damage to the rails, and no deterioration in the geometry. The ESAH-M measuring system measures dynamic three-dimensional acceleration. The video measuring system records the dynamic movements of rails and switch sleepers during the passage of rolling stock. Both of the measuring systems are located off of the turnout, ensuring safe operation and continuous measurement [60].

Kovalchuk et al. [61] presented a system for diagnosing the crossings of turnouts by measuring the transverse profile. This method is based on the use of modern microcontrollers with high technical parameters and the simultaneous use of Internet of Things (IoT) information technology.

Ma et al. [62] presented parameter studies on the surface-initiated rolling contact fatigue (RCF) of turnout rails using a three-level unreplicated saturated factorial design. The results showed that the rail surface-initiated RCF is mainly caused by tangential stress that is high under small creep conditions, normal and tangential stresses that are high under large creep conditions, and normal stress that is high under pure spin creep conditions.

In turn, Wei et al. [63] focused on assessing the degradation of crossings at turnouts via wheelset acceleration measurements. The possibility of evaluating the degradation of crossings at turnouts via axle box acceleration (ABA) was investigated. Information was collected from many sensors: ABA signals, three-dimensional rail profiles, GPS and tachometric records, and both nominal and degraded passages. An algorithm was proposed in order to distinguish the ABA characteristics that are associated with degradation and then assess the conditions of the crossings at turnouts. The types and degrees of degradation can then be assessed based on the spatial distribution and energy concentration of the characteristic ABA signal frequencies.

Pållson [64] also refered to the monitoring of crossings at turnouts and presented a numerical method for optimizing robust geometry. The work presented in [65] provided relevant criteria and explored the basic and multidisciplinary issues concerning the design and practical selection of composite materials in turnout systems. The influence of composite carriers on track geometry (recorded by “AK Car” track control vehicle based on measurement data), track settlement, track dynamics, and acoustic characteristics was highlighted.

In [66], experimental studies were performed on the influence of holes and openings in webs on the strength and ductility of concrete. An et al. [67] referred to the observation and simulation of wheel set acceleration on a railway weld in a high-speed rail system. Irregularities can cause a high amount of force at the wheel–rail contact point and they are considered to be the main cause of the failure of the track structure. In [68], a 3D model of dynamic rolling friction with finite elements (FEs) was presented for a small pitch corrugation test, taking into account the direct and momentary coupling between the contact mechanics and the dynamics of the structure in the vehicle–rail system.

Laser scanning and global navigation satellite system (GNSS) technology were used for the spatial analysis of railway infrastructure condition geometry [69,70,71,72,73,74,75]. In [71], a cost–benefit analysis of Building Information Modeling (BIM) in the railway area was presented, including many railway tracks, stations, telecommunication facilities, infrastructure facilities, railway structures, etc. The experimental results in [69] showed that this method can not only quickly extract linear objects, such as railway tracks and catenary wires but can also detect objects in complex real topologies such as arches and turnouts. Kaewunruen and Lian [76] presented a digital twin aided sustainability-based lifecycle management method for railway turnout systems. The efficiency and effectiveness of their maintenance can be improved by integrating existing practice with Building Information Modelling due to the complexity of railway turnouts. This research established and analysed the world’s first 6D BIM for the lifecycle management of a railway turnout system. The digital twins of a railway turnout in 3D include the scheduling, costs, and sustainability across the whole turnout lifecycle. Maciuk [73,74] noted that GNSS plays an important role in civil engineering and other technical fields. A certain level of accuracy is necessary for solving certain surveying and engineering issues [72,73]. Zhang et al. [75] responded to the required assessment of the relative spatial accuracy of GNSS/inertial navigation systems (INSs) with limited traffic for the measurement of track irregularities.

## 3. Methodology

The following subsections describe, in detail, the surveying method that is based on obtuse crossings and arising from the geometric dependencies in double and outside slip turnouts.

### 3.1. Surveying the Geometric Center Double Slip Turnout—Rkpd_iwew_—With Blades Inside the Quadrilateral

**Figure 2:**
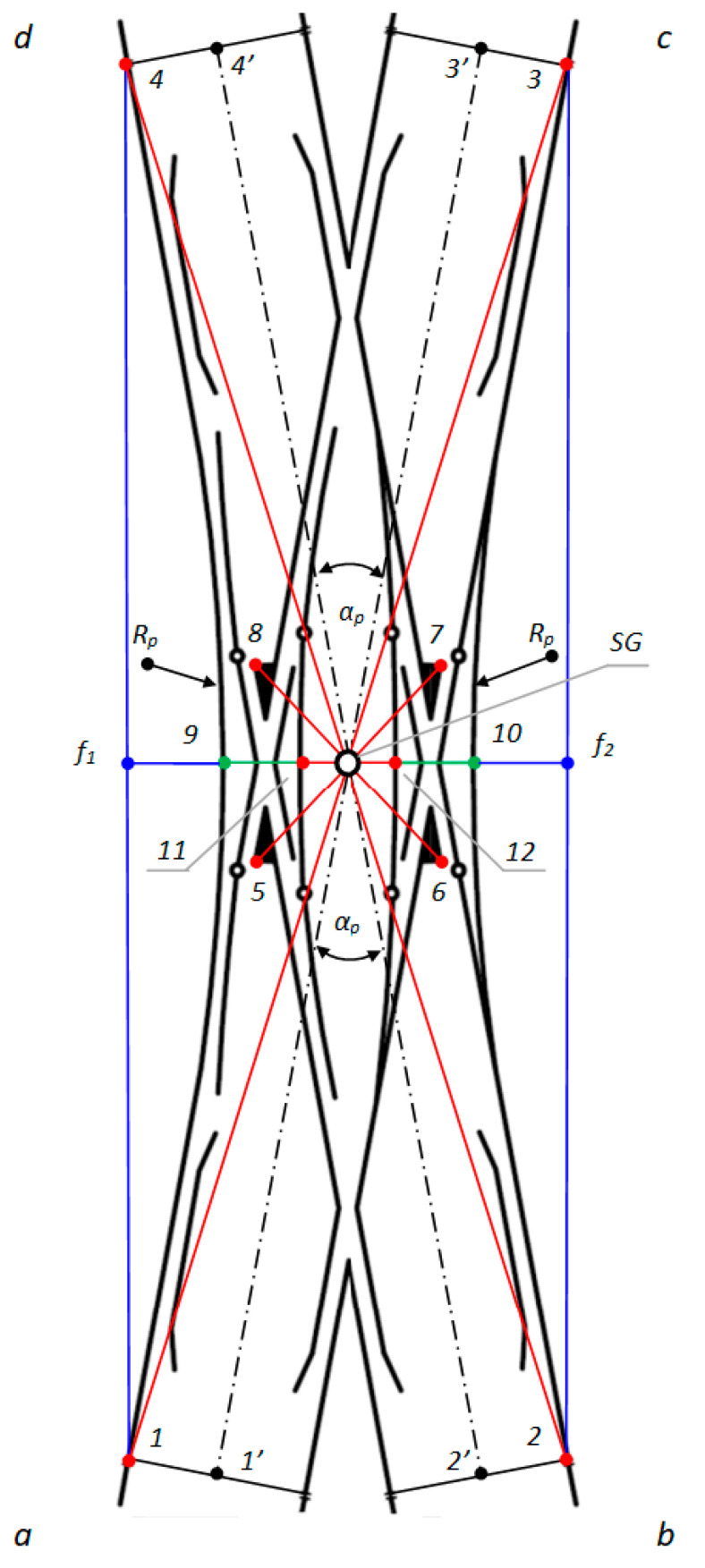
Double slip turnout of Rkpd_iwew_ with blades inside the quadrilateral of the turnout using the Surveying and Monitoring of the Geometric Center of a Double and Outside Slip Turnout (SMDOST) method.

The geometric center of a double slip turnout is the mathematical point of the turnout determined by the intersection of the axes on the main tracks of the turnout (theoretically). In the center of the double slip turnout, there is an obtuse crossing (Figure 1 and Figure 2) that consists of two noses (beaks), a wing rail, and a check rail to ensure that the flange of the opposite wheel is introduced into the correct flangeway (Figure 2). The crossing is the part of the turnout where the rails intersect. Crossings are usually subject to rail wear [13,47,60], which is a result of the friction of the surface caused by the wheels of the rolling stock. The rules for obtuse crossing assembly are defined in [77] and refer to the angular position values of the obtuse crossings, which should be placed exactly opposite each other and parallel.

The following definitions apply to Figure 2:

- SG = the geometric center of Rkpd_iwew_;
- 1, 2, 3, 4 = endpoints of Rkpd_iwew_ (from sides “a”, “b”, “c”, and “d”, respectively);
- 5, 6, 7, 8 = the external endpoints of the obtuse crossing of Rkpd_iwew_;
- 9, 10 = the points of contact connecting the stock rail in a straight line, defining the geometric center of Rkpd_iwew_;
- 11, 12 = the points of contact connecting the blades on the fixed plates in a straight line, determining the geometric center of Rkpd_iwew_;
- 1′, 2′, 3′, 4′ = endpoints of Rkpd_iwew_ on the axis of the main track (from sides “a”, “b”, “c”, and “d”, respectively);
- f_1_ = measuring the versine on chords 1–4;
- f_2_ = measuring the versine on chords 2 and 3;
- α_p_ = main turnout angle—project (between the main track axles); and,
- R_p_ = project radius.

To carry out surveying with reference to obtuse crossings and the resulting geometrical dependencies, the method of measurement was defined together with the measurement points in Rkpd_iwew_, which required the application of a Geodetic Mini Prism (surveying mini prism) for the following:

- The external endpoints of the obtuse crossings of Rkpd_iwew_;
- The points of contact connecting the stock rail in a straight line, defining the geometric center of Rkpd_iwew_;
- The points of contact connecting the blades on the fixed plates in a straight line, determining the geometric center of Rkpd_iwew_; and,
- The endpoints of Rkpd_iwew_.

To date, the designation for the geometric location of the center of the Rkpd_iwew_ turnout has only referred to the endpoints of Turnouts 1–4 (Figure 2). However, to correctly determine the geometric center SG, reference should be made to the following (Figure 2):

1. External points of Obtuse Crossings 5–8 and the following:
  - The points of contact—No. 9 and 10—connecting the stock rail in a straight line, defining the geometric center of Rkpd_iwew_; and,
  - The points of contact—No. 11 and 12—connecting the blades on the fixed plates in a straight line, determining the geometric center of Rkpd_iwew_;
2. Turnout Endpoints 1–4.

If the SG point, with reference to an obtuse crossing (featuring additional points and endpoints of the turnout of Rkpd_iwew_) is unambiguously determined by intersecting straight lines and it is located in one place, then the turnout has a correctly located geometric center SG. Otherwise, it is geometrically irregular.

An important criterion for the analysis and evaluation of the geometric condition of Rkpd is monitoring the horizontal versine of the turnout f_1_ and f_2_ (Figure 2). The conducted research has shown that, in the current practices of Rkpd implementation and exploitation, the monitoring of the horizontal versine value in turnout-diverted tracks is not carried out. However, previous research shows the need to monitor the horizontal versine, whose value reflects the radius of the curve R, which influences the state of the turnout geometric system. On a plane, a circle can be described with a radius R, which is realized on three points. The linear elements of the resulting triangle are the height, which is the horizontal versine of the curve, and the base, which is the measuring chord (Figure 2) [37]. The versine value of the curve is calculated based on the coordinates of the points forming the triangle of the versines, in which the height of the triangle is the versine of the curve [37].

**Figure 3:**
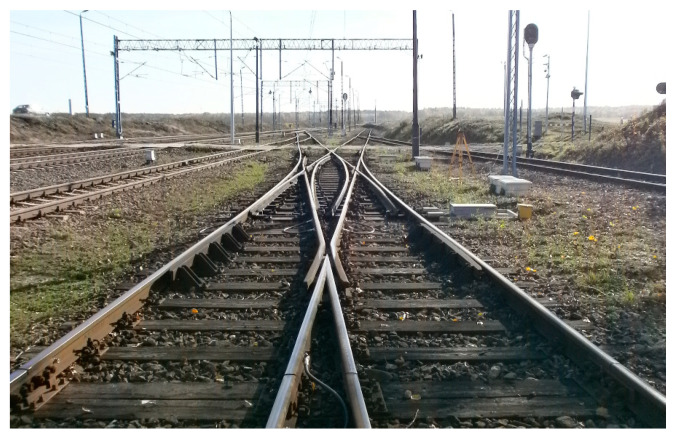
SMDOST-monitored double slip turnout with blades inside the quadrangle Rkpd_iwew_.

Measurements using the SMDOST method were carried out on an exploited double slip turnout with blades inside the quadrilateral Rkpd_iwew_ No. 9 type S49/1:9/190 (Figure 3), which was built on the main additional track (No. 6) assigned to Railway Line No. 131 Chorzów Batory–Tczew in the territory of the Silesian Voivodship, Lubliniecki County, Poland. Additionally, Turnout No. 9 is characterized by the following (Figure 3):

- Zipper closures—locking points in the turnout railway;
- Blade resilience;
- Electrical adjustability;
- Crossings with a forge-welded nose;
- Railway fish plate variety;
- No electric heaters;
- Wooden switch sleepers; and,
- Ballast—natural broken stone.

**Figure 4:**
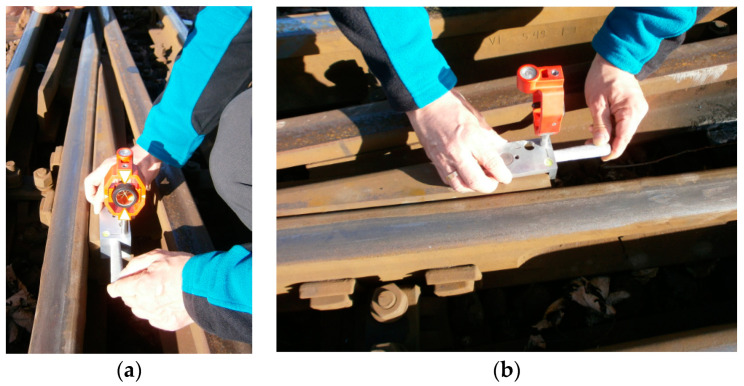
The Magnetic-Measuring Square (MMS) with the surveying GMP111 mini prism located on the external end of an obtuse crossing: (**a**) forward view; and, (**b**) side view.

The measurement Rkpd_iwew_ was performed using MMS with a Leica GMP111 mini prism, prism constant +17.5 mm (Figure 4), and an electronic total station TC407 Leica. The accuracy of the measured points m_p(pom)_ at the Rkpd_iwew_ No. 9 turnout using the polar method m_p(pom)_ was 0.002 m, the line-of-sight error (H_z_-collimation) was 0.0000 ^g^, and the V_z_-Index (Vertical index error) was 0.0000 ^g^. The measuring station of the electronic total station was located in the intertrack space in the middle of the total length of the double slip turnout, outside the infrastructure gauge (structure gauge). The location of the electronic total station (Leica TC407) ensured a uniform and short aiming line for the instrument lengths (line of sight/collimation axis) of all the measuring points. This study excluded jumps in different lengths of the aiming line of the instrument to increase the measurement accuracy. Measurements were taken from one location using the surveying polar method (the surveying method of polar coordinates), and the versine was measured in the outer rails. As a result, an identical accuracy value of m_p_ was obtained for all of the measuring points. The polar method involves measuring the horizontal angle and length of the aiming line of the instrument from the position of the electronic total station—i.e., the center over the surveying matrix point to the turnout measurement points (defined in the SMDOST method) between the side of the matrix and the target axis. Distance and angle measurements were performed simultaneously with a TC407 electronic total station (measurements in one series). The use of MMS with a GMP111 mini prism ensured the station’s correct location on the external endpoints of the measurement: the obtuse crossing, the contacts connecting the stock rails, the contacts connecting the blades on the fixed plates, and the turnout endpoints (Figure 2 and Figure 4).

The MMS with the GMP111 mini prism and the electronic total station provided the coordinates for Points 1–12. The final stage was to obtain the angular and linear values of Rkpd_iwew_, which characterize the turnout geometry. A feature of the MMS is that the vertical axis of its stylus mounting hole coincides with the measuring edge and with the axis of the surveying stylus and the geometric center of the GMP111 mini prism. As a result of the neodymium magnets built into its structure, the MMS was stabilized immovably for the Rkpd_iwew_ steel sections, providing a visualization at each measurement point (Figure 4).

To date, only the endpoints of Rkpd turnout were used to determine the geometric location of the center of the turnout. These measurements were carried out through:

- The points of intersection (cuts) using the measuring lines (cord measuring) as diagonals. However, the components of the steel sections as a turnout structure run at different heights, thereby causing the measuring line to cut incorrectly on the horizontal plane (e.g., rails and check rails). In addition, weather conditions influence the measurement results (wind) and string that is too long can cause sagging and suspension from the turnout’s structural elements. The manoeuvring of the measuring line on a specific turnout reflects the accuracy and safety of the work (the measuring line can fall and hook between the blade or stock rail). This makes it impossible to quickly remove this line from the exploited turnout;
- The point of intersection of straight lines using ranging poles (survey poles) located at the axis of the turnout endpoints and delimiting the points of intersection acting as the designated centers of the turnout. This method required setting a minimum of five surveying poles directly at the Rkpd, which is a reference for the accuracy of the point to be determined. In the situation of an incoming train, the surveying poles had to be removed quickly, and after the train had left, the measurement process had to be repeated;
- Using a traditional surveying prism or a mini-prism with a survey pole and the surveying pole tip as a standard surveying solution and applying both to the steel elements of the turnout structure for which traditional survey poles and surveying pole tips do not provide correct point of destination signaling. The traditional solutions used so far resulted in signalling errors depending on the non-verticality of the survey pole and the surveying pole tips with the surveying prism; not locating the prism at a height of 14 mm below the top rolling surface of the rail head (crown of the rail) induced a signalling error for the destination point on the steel turnout sections; and,
- Measurements of the main length elements via surveying tape. The operation of the developed length of the surveying tape with a minimum length of 33.230 m (turnout length) reflected the measurement accuracy and safety of the works.

So far, there have been no measurements of the versines on the diverted tracks of turnouts. As a result of introducing the SMDOST and MMS methods, the measurement process was optimized with an increase in measurement accuracy and safety, not only for the surveying employee’s presence on the exploited turnout but also the correct determination of the position of the geometric center of the double slip turnout (and the outside slip turnout), as well as ensuring the geometric conditions of the circular arcs of the diverted track. The use of SMDOST and MMS is related to the steel elements of the turnout structures and the availability of specific turnout characteristics. SMDOST relates to:

- The external endpoints of the obtuse crossing (which have not yet been defined);
- Extra points such as the points of contact connecting the stock rail and the points of contact connecting the blades; and,
- The turnout endpoints.

The resulting network of straight lines marks point SG in one place by intersecting the lines forming a correctly defined and situated geometrical center SG. Failure to meet one of the conditions for the reference network of measurement points or when reference is made to such points but SG is not in one place results in a geometrical irregularity (geometrical anomaly).

The accuracy of the measured points is defined by the following:

- The location of an electronic total station in the coordinate system. The measurements were carried out using a single setup (station, location) in the so-called local coordinate system defined by the instrument, thereby ensuring a common coordinate system and the uniform accuracy of measurements for the whole turnout. The measuring station’s (measuring setup) electronic total station was located in the middle of the total length of the double slip turnout outside the infrastructure gauge (structure gauge) in the intertrack space. Because the measurements carried out in the coordinate system were defined by the instrument itself and not by the state coordinate system, the effect of the electronic total station’s location was delimited;
- State of instrumental errors. Errors were eliminated by calibrating the instrument and using the measurement methods;
- The influence of atmospheric conditions, which were limited by the application of so-called atmospheric corrections (e.g., air temperature and pressure during the measurements);
- Signalling the measuring point at the turnout. The turnout is characterized by a large number of steel elements with characteristic profile shapes. As a result of the integration of the geodetic mini prism with the MMS (equipped with neodymium magnets), the turnout was stabilized directly above the measuring points, followed by the verticality and correct signalling of points at a height of 14 mm below the top of the rolling rail head or the lower edge of the steel elements of the turnout (e.g., wedges on the obtuse crossing) together with the provision of an aiming direction (pointing line, sight line). The height of the Leica GMP111 geodetic mini prism integrated into the MMS was 10 cm; and,
- The accuracy of the distance and angle measurement is a characteristic feature of a stationary electronic total station, as defined by ISO 17123: accuracy standard deviation H_sd_—horizontal direction, V_sd_—vertical angle/zenith angle (acc. to ISO 17123-3) is 7″ (20^cc^); electronic Distance Measurement—EDM measuring program IR_Dokł/IR_Fine (standard deviation acc. to ISO 17123-4) is 2 mm + 2 ppm [78].

The accuracy of the measured points m_p(pom)_ in the turnout Rkpd_iwew_ was 0.002 m using the SMDOST method and MMS integrated with the Leica GMP111 geodetic mini prism and the Leica TC407 electronic total station. This accuracy could be increased by using an electronic total station and high accuracy surveying prisms integrated with MMS and SMDOST.

MMS is also compatible with other surveying prisms that can be used depending on the measurement purpose. The increasing use of railway infrastructure for passenger and freight transport makes it necessary to maintain the large capacity and safe maintenance of railways. Thus the application of the SMDOST and MMS method is of crucial importance to monitor turnouts in the most optimal, safe, and effective way.

### 3.2. Surveying the Geometric Center Outside the Slip Turnout—Rkpd_izew_—With Blades Outside the Quadrilateral

**Figure 5:**
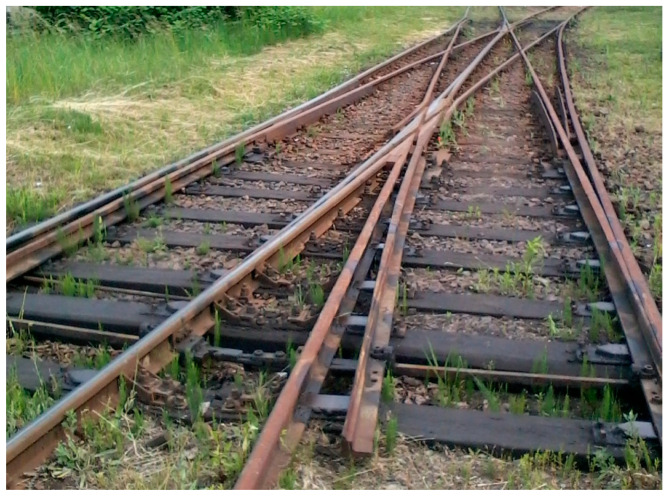
Outside slip turnout with blades outside the quadrilateral turnout of Rkpd_izew_.

Surveying the location of the geometric center SG of the defined SMDOST method is also used in the same way to monitor the outside slip turnout with blades on the outside of the Rkpd_izew_ quadrilateral (Bäseler system turnouts; in practice, called outside slip turnout). Rkpd_izew_ is characterized by its complex construction, featuring arches of 300 m or more. The blades of these turnouts are located in front of the crossings due to its longer tangential arches (Figure 5).

**Figure 6:**
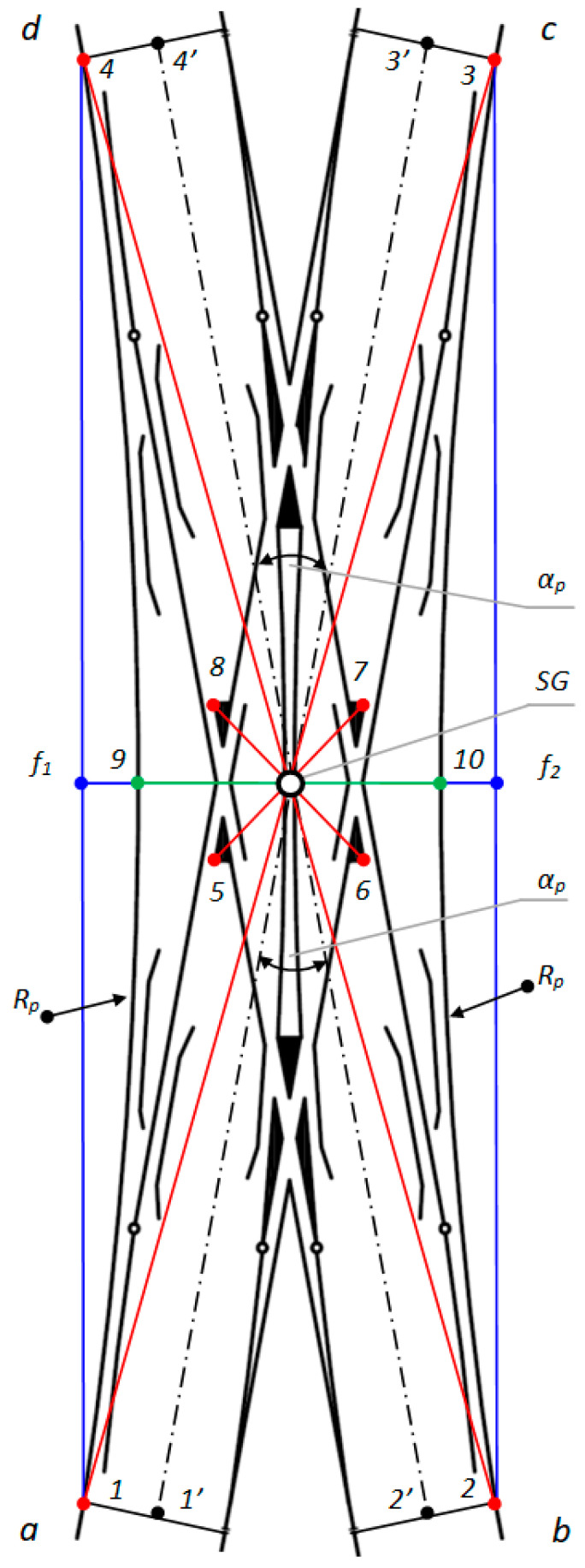
Outside slip turnout for Rkpd_izew_ with blades on the outside of the quadrangle using the SMDOST method.

The measurement points for Rkpd_izew_ were also defined, requiring the application of a surveying mini prism in the following areas, in order to carry out the SMDOST method of surveying (Figure 6):

- The external endpoints of the obtuse crossings of Rkpd_izew_;
- The points of contact connecting the stock rail in a straight line, thereby defining the geometric center of Rkpd_izew_; and,
- The endpoints of Rkpd_izew_.

The following definitions apply to Figure 6:

- SG = geometric center of Rkpd_izew_;
- 1, 2, 3, 4 = endpoints of Rkpd_izew_ (from the sides “a”, “b”, “c”, and “d”, respectively);
- 5, 6, 7, 8 = external endpoints of the obtuse crossing of Rkpd_izew_;
- 9, 10 = the points of contact connecting the stock rail in a straight line, thereby defining the geometric center of Rkpd_izew_;
- 1′, 2′, 3′, 4′ = endpoints of Rkpd_izew_ in the axis of the main track (from the sides “a”, “b”, “c”, and “d”, respectively);
- f_1_ = measuring the versine on chords 1–4;
- f_2_ = measuring the versine on chords 2 and 3;
- α_p_ = main turnout angle—project (between the main track axles); and,
- R_p_ = project radius.

To determine the point of the geometric center SG, reference should be made to the following (Figure 6):

1. The external points of Obtuse Crossings 5–8 and the points of contact connecting the stock rail in a straight line (No. 9 and 10), defining the geometric center of Rkpd_izew_; and,
2. The turnout Endpoints 1–4.

If the point SG with reference to obtuse crossings and the points of contact connecting the stock rail in a straight line and the turnout endpoints of Rkpd_izew_ is unambiguously determined by the intersecting straight lines and is located in one place, then the turnout has a correctly located geometrical center SG. Otherwise, it is a geometric anomaly of the Rkpd_izew_ turnout.

## 4. Results and Discussion

### 4.1. Structure of the Number of Derailments and Safety Status

According to requirements of the European Railway Agency (ERA) and of the Office of Rail Transportation (UTK) for the railway network managed by PKP PLK S.A. (Polish State Railways Polish Railway Lines Joint Stock Company, in Polish: Polskie Koleje Państwowe Polskie Linie Kolejowe Spółka Akcyjna, PKP PLK S.A.), the applied classification of railway accidents includes [79]:

- collisions;
- derailments;
- accidents at level crossings and pedestrian crossings;
- accidents including persons outside level crossings and pedestrian crossings (excluding suicides);
- rolling stock fires; and,
- other accidents.

**Figure 7:**
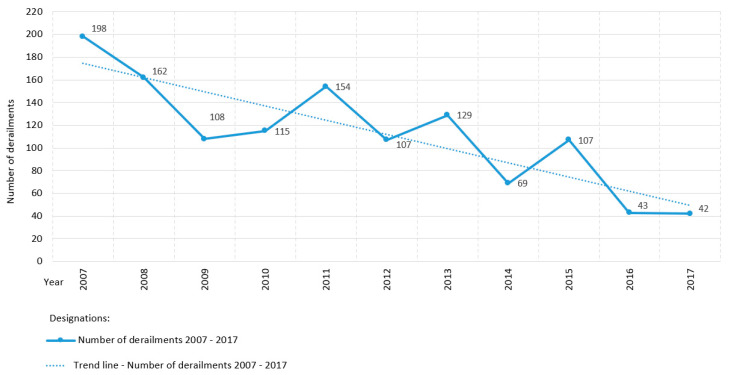
State of derailments on the railway network managed by PKP PLK S.A. in Poland in the years 2007–2017.

Figure 7 shows the state of derailments on the railway network managed by PKP PLK S.A. in Poland from 2007 to 2017. Derailments are a component of an accident, defined as an unintentional sudden event or a sequence of such events involving a railway vehicle, yielding negative consequences for human health, property, and/or environment [1]. The trend line demonstrates the phenomenon of derailments in the period 2007–2017 to be in a descending orientation, progressing over time proportionally (Figure 7). The average number of all derailments in the period of 2007–2017 in Poland is 112.2.

**Figure 8:**
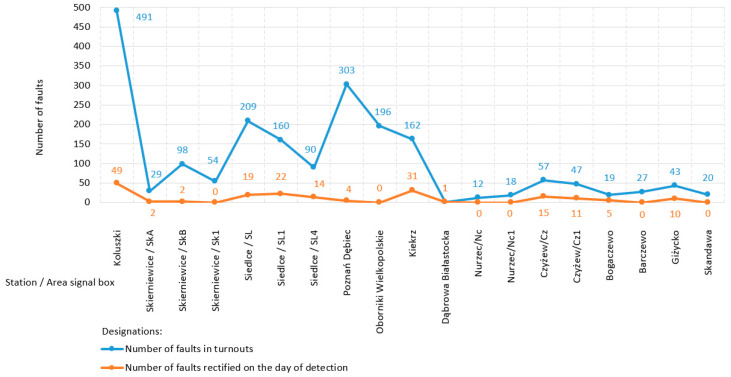
Status of the number of faults in turnouts in selected stations/area signal boxes.

The state of the number of faults at the turnouts of the selected stations/area signal boxes (signal box, area under control, railway control command, and signaling building) of the railway network in Poland is illustrated by the graph presented in Figure 8. This graph includes 19 stations/area signal boxes with information about the number of faults at the turnouts in each of them and the number of faults rectified on the day of their detection (direct intervention) [80]. The safety condition at the turnouts was unsatisfactory, depending on the type of fault requiring direct or timely intervention with previous protection. The average time need to remove the remaining (timely) faults was 10 days.

**Figure 9:**
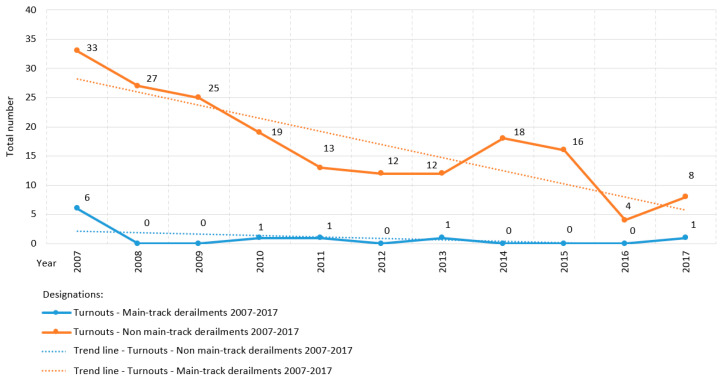
State of derailments at Canadian turnouts in 2007–2017.

Of all the railway networks in the world, Canada’s is the third largest and transports the fourth largest volume of goods) [81]. Investigations on rail modes explore a wide variety of subjects, such as operational decision making, risk management, component failure, supervision, metallurgy, and track train dynamics [81]. The graph presented in Figure 9 shows the state of derailments at turnouts on the North American continent in Canada, providing a summary of the number of derailments during the period of 2007–2017:

- Main-track derailments; and,
- Non main-track derailments.

Non main-track derailments on turnouts are more common than main-track derailments. The trend line of derailments in both graphs over the years 2007–2017 presents a gradual decreasing trend (Figure 9). However, this trend indicates the need for further open research to guarantee the quality of safety at turnouts. This is also confirmed by the studies that were presented by Batarlienė in [82], demonstrating that transport, due to its specificity and risk, must be precisely controlled, regulated, and operated. The analyses of rail transport and the probability of accidents suggest that although all accidents are decreasing in number, accidents involving dangerous goods occur especially frequently.

The statistical data on derailments confirm the claim in [83] that derailments are common. Mistry et al. in [84] concluded that railway point-operating machine (POM) failures are considered to be critical failures of a rail network system. Signaling equipment and turnout failures account for 55% of all railway infrastructure component failures, leading to delays, costly repairs, and potentially hazardous situations. In [83], Dindar et al. presented an integrated approach for handling the many different risks that arise from various sources in railway turnout systems by identifying suitable multi-disciplinary risk analysis methods for these complex systems. The authors referred to the various qualitative- and quantitative-based risk analysis methods proposed to fully understand a number of technical phenomena, such as ageing, degradation, and signalling faults, in a railway turnout system. The authors found that one of the fundamental reasons for derailment incidents at turnouts may be the fact that the railway industry pays little attention to the risk elements of railway turnouts. Not only is the prediction of such elements complex and difficult but it also requires a comprehensive range of applications and a well-designed geographic information system. In [85], a new stochastic mathematical prediction model was established based on a hierarchical Bayesian model (HBM), to better address unique exposure indicators in segmented large-scale regions.

Importantly, the continuous improvement and optimisation of the level of safety performance of railway undertakings and infrastructure management is an essential component in the field of rail transport safety management. Measurement innovation in railway infrastructure safety also entails areas of measurement work (measuring methods, techniques, and instruments) that are interdependent and complementary based on the SMDOST method with MMS.

### 4.2. Case study and Field Measurements

This section presents the results and discussion of the application of the SMDOST method to a double slip turnout. Attention was paid to the correctness of the geometric relationships of the monitored turnout. Reference was also made to the geometric irregularities of the turnouts. There is a strict dependence between correctly determining the position of the geometric center point SG of a double slip turnout and other structural elements. The value of the horizontal versine f_i_ indicates the condition of the R_i_ rays of the diverted track. The present research focused on real Rkpd_iwew_ measurements, demonstrating and confirming that monitoring the condition of turnout geometry plays a key role in the safe operation of rail transport, essentially affecting quality, comfort, and safety. The presented SMDOST method confirms the its applicability. This method allows one to correctly determine the position of the Rkpd geometric center point along with other turnout geometric dependencies. Referring to the geometric relationships of a double slip turnout, Rama and Andrews [22] rightly noted that turnouts and diamond crossings are multi-component systems and that to actively prevent failures and undesirable consequences, it is necessary to anticipate problems at the component level.

Table 1 presents the results of the measurement of the Rkpd_iwew_ No. 9 ab/cd turnout type S49/1:9/190 using the SMDOST method. Calculations were made for the values of the existing rays of the diverted track in the directions of the “ad”–R_iad_ radius and the “bc”–R_ibc_ radius. Theoretically, the values of the R_iad_ and R_ibc_ radii should be identical and consistent with the value of the projected radius, R_p_ = 190 m; however, in a deformed turnout, a discrepancy may occur (analogous to the main angle of the turnout α_p_).

The relationship between the radii of the R_i_ curve and the arrows f_i_ can be determined by Equation (1):

(1) $$R_{i}=\frac{a\;\cdot b}{2\;\cdot f_{i}},$$

where R_i_ is the curve radius, f_i_ is the horizontal versine of the curve, and a and b are the distances between points. The obtained value of the first radius of the diverted track “ad” is R_iad_ = 223.804 m ≈ 225 m. The value of the second diverted track “bc” is R_ibc_ = 223.655 m ≈ 225 m (Table 1). The measurements showed the difference in the existing radius R_i_ = R_iad_ = R_ibc_ to the projected radius R_p_, whose difference is 35 m. Table 1 shows the differences between the linear and angular values of the covered turnout.

According to the recommendations of the Id-4 regulations [23], the versine is not measured in slip turnouts (double and outside slip turnout and single slip turnout). Instruction Id-4 [23] defines the methods, rules of measurements, dates for performing visual inspections and technical studies, and the rules for performing repairs of all the turnouts under exploitation. According to the author, this record is controversial, because, with the value of an existing versine, one can determine the existing state of the diverted track geometry. The tests using the SMDOST method revealed the abnormal curvature of the turnout-diverted track and a change in the values of its radii. The state of the curvature of the diverted track is an essential element not only for the correct maintenance of operation works, but also for the implementation of work–turnout assembly (monitoring the correctness of the turnout assembly). Thus, not only the choice of the permissible track gauge and cant parameters, but also the permissible speed on the diverted track depend on the state of the curve radius of the diverted track. The following are also important: the speed of the wear and tear of the structural elements, changes in the values of the track gauge and cant parameter (and, thus, the derivative parameters), and the wear and tear of the headland switch sleepers. The anomalies resulting from both the wrong location of the geometric center point of the SG turnout and the condition of the diverted track contribute to the impact of rolling stock running turbulence. In this case, running the rolling stock on turnout tracks whose geometrical parameters are not adjusted to the specified speed causes vibrations due to accelerations (excessive centrifugal forces).

**Figure 10:**
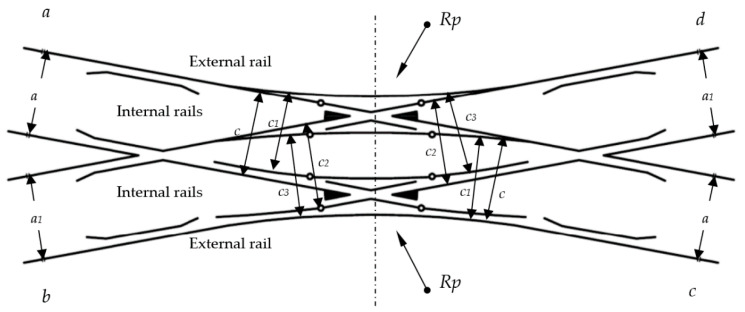
Measurement of the track gauge and cant parameter at the point cross-sections.

The SMDOST method used for the double slip turnout No. 9 ab/cd type S49/1:9/190 showed that the SG point was not clearly defined by the intersecting straight lines and not located in one place. The geometric center of double slip turnout SG, as the point determined by the components of the obtuse crossings and their geometric relationships, does not coincide with the point of the geometric center related to the endpoints of the turnout and their geometric relationships. In the monitored turnout, there was an irregularity in the position of the point of the geometric center SG. Four points were ultimately obtained, which formed a quadrilateral. The coordinates of the center of gravity of the quadrilateral were then calculated and considered as the final operational position of the geometric center SG of the double slip turnout. The coordinates were transformed using the Helmert method and declared as adjustment points (point of minor control, register mark) No. 2 and 4 on the main track for the direction “bd”—dimension a_1_ (Figure 2 and Figure 10).

In Figure 10, the following definitions apply:

- a track gauge and cant–turnout pre-blade contact for turnout side “a” and “c”;
- a_1_ track gauge and cant–turnout pre-blade contact for turnout side “b” and “d”;
- c track gauge and cant of the switch (on the first fixed plate) of the internal rails—the “a” and “c” sides of the turnout on the main track;
- c_1_ track gauge and cant of the switch (on the first fixed plate) of the external rails—the “a” and “c” sides of the turnout on the diverted track;
- c_2_ track gauge and cant of the switch (on the first fixed plate) of the internal rails—the “b” and “d” sides of the switch on the main track;
- c_3_ track gauge and cant of the switch (on the first fixed plate) of the external rails—the “b” and “d” sides of the switch on the diverted track;
- a, b, c, d turnout sides; and,
- R_p_ project radius.

The study carried out on the values of the track gauges in Sections No. 2 and 4 showed stability, amounting to 1435 mm (Table 2). The position of the exploitation point SG in relation to the actual state differed by 0.011 m. The specific dimensions presented in Table 2, Table 3 and Table 4 should be interpreted with a prefix of 14 mm.

In the turnout pre-blade contact of the Rkpd_iwew_ No. 9 ab/cd type S49/1:9/190, the track gauge and cant at the a and a_1_ points were periodically monitored (Figure 10):

- a Track gauge and cant–turnout pre-blade contact on turnout side “a” and “c”; and,
- a_1_ Track gauge and cant–turnout pre-blade contact on turnout side “b” and “d”.

The proper condition for the track gauge parameters of Rkpd_iwew_ type S49/1:9/190 at turnout pre-blade contact in sections a and a_1_ was 1435 mm, and the permissible deviation was from +8 to −4 mm. The deviation in the permissible mutual height position of the rails (track path cant) ranged from +12 to −12 mm [23]. The track gauge of the turnouts is the distance between the internal surfaces of the rails measured 14 mm below their running surface. The track cant in the turnout is the difference in the height of the rails in one cross section. The position of the track in the cross-section is the measured difference in the height of the rails in one section of the track in the vertical plane [24,25], taking international law into account.

Table 2 presents the results of the monitoring track gauge and cant values directly in front of the turnout pre-blade contact of Rkpd_iwew_ turnout 9 at points a and a_1_. The investigations showed the stability of the main track at the cross-sections of the a_1_ points in the “bd” direction, where the value of the track gauge parameter was 1435 mm (Figure 10). In the section of the a points in the “ac” direction, the value of the track gauge parameter exceeded the permissible deviation of the maximum (1445 mm).

The Rkpd_iwew_ geometric condition was additionally confirmed by monitoring four turnouts within an obtuse crossing for the track gauge and cant parameters (Figure 10). The principles of measurement at the characteristic points of the railway turnouts and diamond crossings and the correctness of the measurements are presented in [33,38], paying particular attention to Rkpd, as well as the measurements of turnout geometry at characteristic points, including within crossings. In the Rkpd_iwew_ switch zone, for both the “ab” and “cd” sides, sections were measured at the following points (Figure 10):

- c The track gauge and cant for the switch (on the first fixed plate) of the internal rails—the “a” and “c” sides of the turnout on the main track;
- c_1_ the track gauge and cant for the switch (on the first fixed plate) of the external rails—the “a” and “c” sides of the turnout on the diverted track;
- c_2_ the track gauge and cant for the switch (on the first fixed plate) of the internal rails—the “b” and “d” sides of the switch on the main track; and,
- c_3_ the track gauge and cant for the switch (on the first fixed plate) of the external rails—the “b” and “d” sides of the switch on the diverted track.

At the double slip turnout, measurements were carried out independently for turnout sides “ab” and “cd.” The measurements of the track gauge parameter also included the track cant parameter.

The proper conditions of the track gauge for the Rkpd_iwew_ type S49/1:9/190 in the switch zone of the main track in cross-sections c and c_2_ were 1435 mm, while, for the diverted track in sections c_1_ and c_3_, they were 1443 mm [23]. The exploitation speed on the main and diverted tracks covered by the turnout measurements was *V* = 40 km/h. The permissible deviation of the track gauge parameter on the main track for dimensions c and c_2_ ranged from +8 to −4 mm. For the dimensions of the parameter track gauge on the diverted track c_1_ and c_3_, the permissible deviation ranged from +14 to −4 mm. The permissible track gauge deviations for the main track depend on the speed, whereas, on the diverted track, they depend on the value of the curve radius [23]. The permissible canting deviations are dependent on the maximum speed at the turnout. This deviation for the monitored turnouts ranged from +12 to −12 mm [23].

Table 3 presents the results of the measurements of the track gauge and cant status in the Rkpd_iwew_ switch at points c, c_1_, c_2_, and c_3_ under the periodic monitoring of turnout No. 9 type S49/1:9/190. The results show that the values remain within the limits of acceptable deviations, for both the main track and the diverted track.

**Figure 11:**
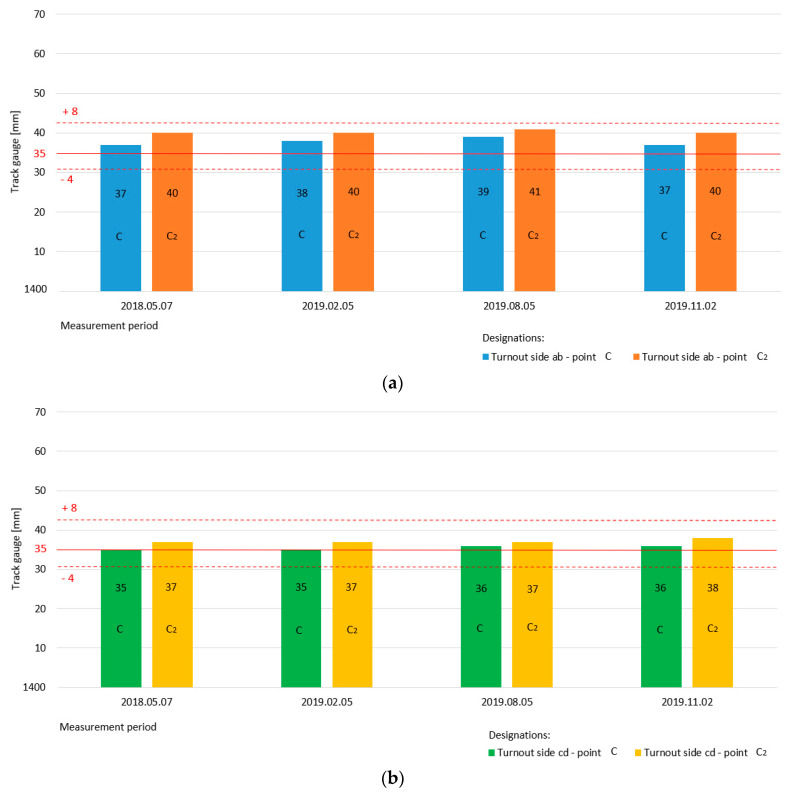
The track gauge of the main track for turnout number 9: (**a**) turnout side “ab” (measurement at c, c_2_); (**b**) turnout side “cd” (measurement at c, c_2_).

Figure 11 shows a graphical interpretation of the state of the parameter track gauge for the main track-switch Rkpd_iwew_ No. 9 for turnout side “ab” (measurement at point c, c_2_) and turnout side “cd” (measurement at point c, c_2_). The specific dimension of the track gauge width in section c and c_2_ is 1435 mm, which is marked by a horizontal continuous red line. The red dotted lines on the diagram define the permissible upper and lower limit values for the width parameter track gauge on the main track, which are +8 and −4 mm, respectively, for dimensions c and c_2_.

The diagram presented in Figure 11a shows the state of the track gauge parameter main track on turnout side “ab”, as well as the measurements made for point c and c_2_ monitored in four measurement periods. The value of the width parameter is within the permissible deviations. The diagram illustrated in Figure 11b also shows the parameter track gauge on the main track for turnout number 9 but for side “cd” (measurements at point c, c_2_). The value of the width parameter track gauge is also within the permissible deviations.

**Figure 12:**
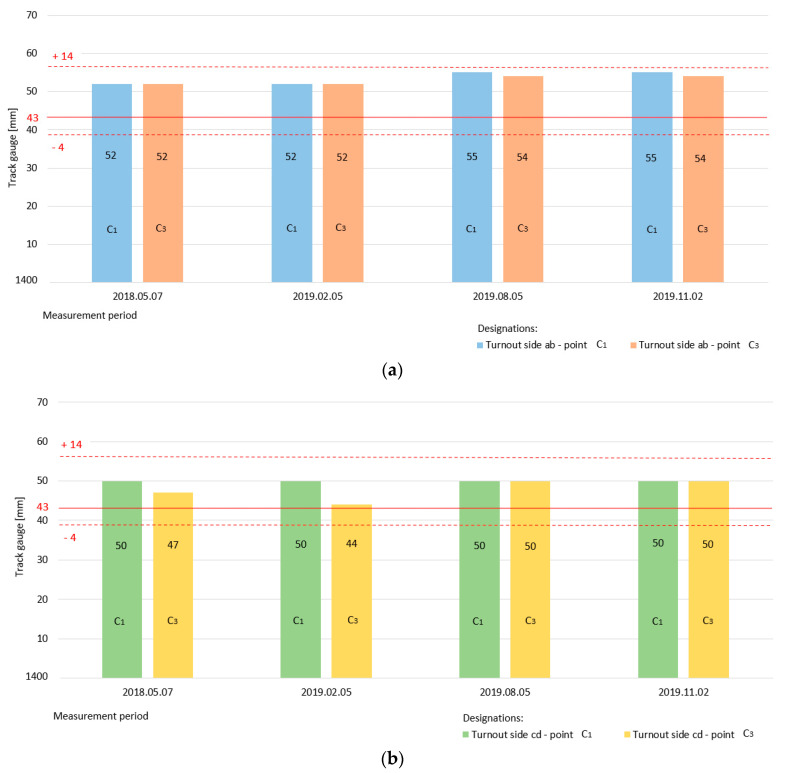
The track gauge of the diverted track in turnout number 9: (**a**) turnout side “ab” (measurement at c_1_, c_3_); (**b**) turnout side “cd” (measurement at c_1_, c_3_).

Figure 12 shows a graphical interpretation of the state of the width parameter track gauge on the diverted track– switch of Rkpd_iwew_ No. 9 for both turnout side “ab” (measurement in point c_1_, c_3_) and turnout side “cd” (measurement in point c_1_, c_3_).

The specific track gauge dimension in section c_1_ and c_3_ is 1443 mm, which is marked by a horizontal continuous red line. The red dashed lines in the diagram are the permissible upper and lower limit values of the diverted track parameter track gauge for values of +14 and −4 mm.

The value of the track gauge parameter is still within the limits of permissible deviations for both turnout side “ab” (Figure 12a) and turnout side “cd” (Figure 12b). The specific dimensions of the track gauge parameter on the diverted track are larger than those of its counterpart on the main track, with a noticeable trend towards the upper limit.

There is a noticeable difficulty in maintaining the correct dimensions for the track gauge and cant of the main and diverted tracks, particularly on the diverted tracks of the arches. This is confirmed by the results of the tests that are outlined in Table 4, which included monitoring the state of the track gauge and cant parameter values in the switches of four additional S49/1:9/190 Rkpd_iwew_ turnouts at points c, c_1_, c_2_, and c_3_.

Each of the four monitored turnouts exceeded the permissible deviations of the track gauge parameter values on the diverted tracks (red marking). The maximum value at point c_1_ was reached at 1478 mm when the specific dimensions should be 1443 mm. However, there was a positive tolerance of 1443 + 14 = 1457 mm. The excess beyond the proper value, including the permissible deviation, was 21 mm (Table 4). There was a significant difficulty in maintaining the correct track gauge parameter dimensions and, therefore, in the track cant in the Rkpd switch diverted track.

**Figure 13:**
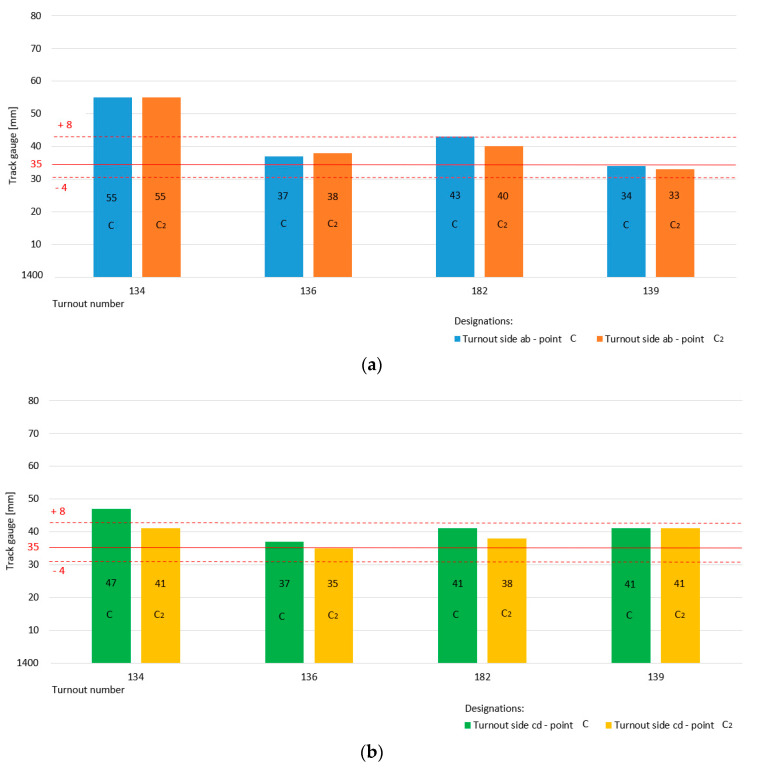
The track gauge of the main track in turnout numbers 134, 136, 182, 139: (**a**) turnout side “ab” (measurement at c, c_2_); (**b**) turnout side “cd” (measurement at c, c_2_).

**Figure 14:**
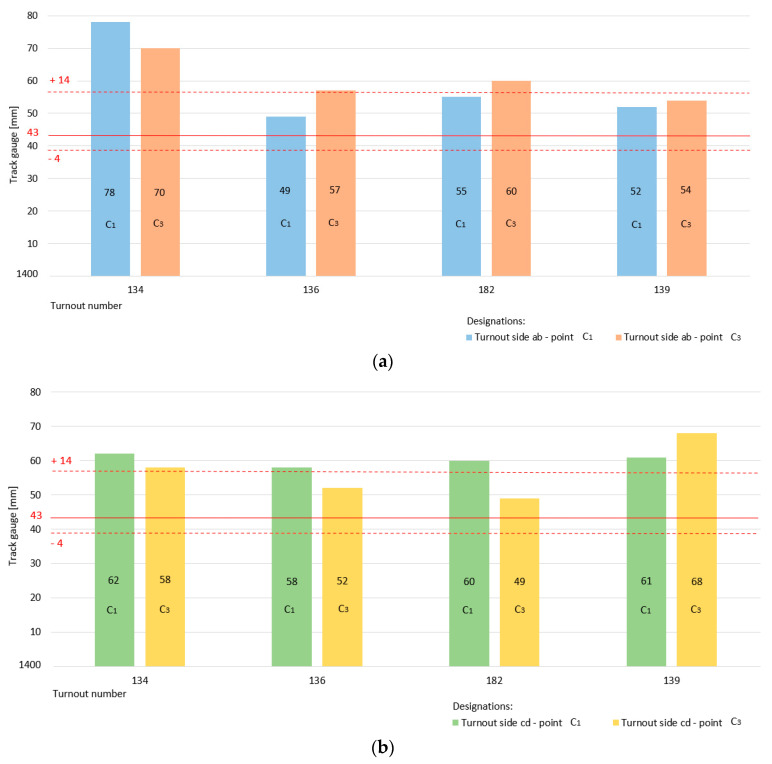
The track gauge of the diverted track in turnout numbers 134, 136, 182, and 139: (**a**) turnout side “ab” (measurement at c_1_, c_3_); (**b**) turnout side “cd” (measurement at c_1_, c_3_).

A graphical visualization of the status of the track gauge parameters monitored at the four additional turnouts No. 134, 136, 182, and 139 is shown in Figure 13 and Figure 14. Figure 13 presents the state of the track gauge parameter on the main track for turnout side ab (measurement at point c, c_2_) and turnout side cd (measurement at point c, c_2_). The specific dimension is 1435 mm marked with a red horizontal continuous line, while the dashed lines determine the permissible upper and lower limit values. For turnout No. 134, the value of the track gauge parameter was exceeded above the upper permissible deviation.

Figure 14 refers to the state of the track gauge parameter on the diverted track in the turnouts monitored at points c_1_, c_3_ for turnout sides ”ab” and ”cd”. The red lines indicate the correct value and the intermittent permissible deviations. For turnout side ab in turnout 134 and 182, the upper limit deviation was exceeded, while turnout side ab in turnout 136 was already at its limit value (Figure 14a). For turnout side cd in all additional turnouts, the upper limit deviation was exceeded. On the diverted track turnout, the state of the track gauge parameter was exceeded above the upper permissible deviation (Figure 14b). The consequence of this condition is a change in the state of the diverted track radii and the position of the geometric center point SG of the double slip turnouts.

Hegedűs et al. [6] rightly stated that turnouts operate under difficult conditions and that their reliability requirements are high due to safety and economic factors. Thus, as Aniołek and Herian [7] emphasize, the problem of the durability of turnouts and rails remains open to technological research to determine their structural relationships and mechanical characteristics. The complex and responsible construction of railway roads involves turnouts. In [86], Marx states that professional turnout maintenance requires specialist knowledge for which there is little literature available outside the Deutsche Bahn regulations in Germany. Indeed, before 1994, there was no training programme available. Previously, this knowledge was obtained almost exclusively obtained from turnout manufacturers, but this type of information was only stationary and not geared to maintenance. Complex and intricate turnout systems that include, among other things, slip turnout (double and outside slip turnout) occupy a special position in the field. Knowledge of these systems already resembles a kind of secret science. Rosiński and Michowski [9] observed that the complex physical phenomena occurring within the switch, connected with the significant forces acting between the moving vehicle and the track, require special attention for turnouts.

**Figure 15:**
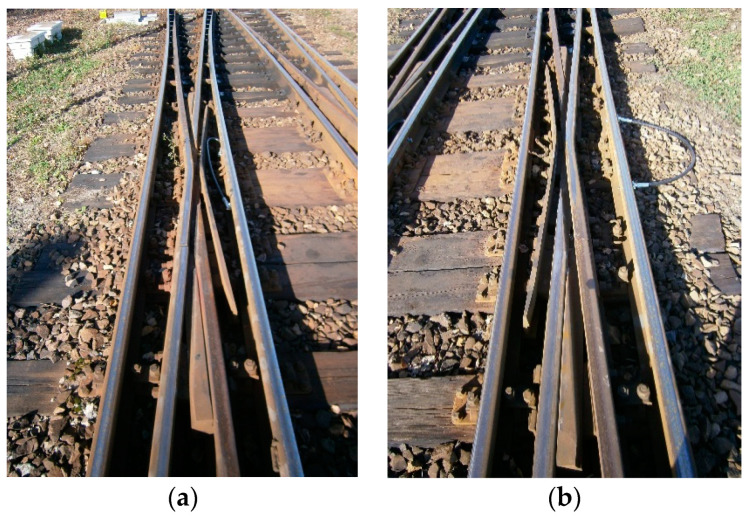
Ballast condition at Rkpd_iwew_ No. 9: (**a**) left-hand obtuse crossing; and, (**b**) right-hand obtuse crossing.

At the same time, wear and tear [13], train axle load [14], directions of the running of the rolling stock, dynamic loads influenced by the profiles of the rolling stock wheels [15], the quality of the rolling stock crossing, the total process of the wheel passing through the turnout [16], the type of crossing and the type of turnout, the interactions between the wheel–rail contact and damage to crossings and turnouts (material, number, etc.) [17], the profiles of the circle elements of the crossing [17,18,19], switch sleeper stiffness, ballast condition, and the elastic properties of the turnout support structure [20,21] all have an impact on the efficiency, effectiveness, and performance of the turnout and its components. Rail safety depends on a number of factors. In particular, the technical conditions of the railway infrastructure influence safety, of which turnouts are an important element. In [87], Kaewunruen et al. highlight that an accurate risk assessment is vital for track components (e.g., embedded anchors, the failure modes of which are dependent on time). Therefore, the time frames in which component risks transition to different risk categories should be determined. In [7], Aniołek and Herian presented a study on burdening and wearing railway switches under exploitation conditions and the materials that were applied for their construction. The authors concentrated on the dynamic loads of a right-railway single point turnout and found that the first dynamic loads occur when the wheelset enters the switch. The highest dynamic loads occur when the wheelset enters the crossing. The condition of the ballast on the Rkpd_iwew_ No. 9 obtuse crossing is a significant element, which is directly reflected in the condition of the railway track–permanent way and turnout structure (Figure 15).

**Figure 16:**
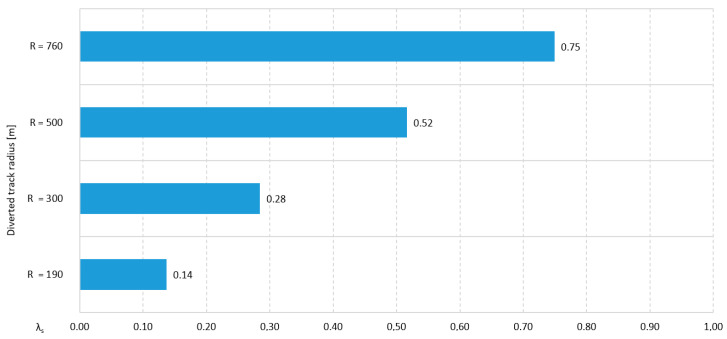
Effects of the curves of the diverted tracks on the service life of the rail.

This ballast is a layer of compacted natural broken stone whose purpose is to provide support and transfer the pressure transmitted by the switch sleepers to the subballast (formation) and drainage layer. Rkpd_iwew_ No. 9 uses Skl-type semi-elastic fastenings (halfelastic fastenings) as screw-and-elastic fastenings that reduce partial vibrational damping. The high dynamic loads acting on the elements of the turnout, which change over time, lead to significant changes in the properties, faster abrasive wear, and local plastic deformation of the material in the rolling layer of the rail sections. Every change in the crossing speed of rolling stock definitively changes the characteristics of the dynamic load, especially when the speed increases [7]. The degradation of the turnout structure, especially in the area of the obtuse crossing and diverted track, accelerates much faster than on the main tracks. This occurs due to the greater dynamic impacts of rail vehicles in places of discontinuous rails. This phenomenon is reflected by the change in the position of the SG turnout’s geometric center. The complexity of turnouts entails the existence of moving elements, many stiffness zones that cause increased dynamic effects, and a weave of dimensions of the parameter value of the track gauge and flangeway, which has great influence on the wheel running angles (the wheel angle of attack). These features make turnouts less durable than the adjacent railway tracks [8]. The deteriorating state of the track gauge parameter within the obtuse crossing for both the main and diverted track is shown in Figure 11 and Figure 12 for turnout No. 9 and for additional turnouts in Figure 13 and Figure 14. The durability of turnouts depends on the durability of the rails (service life of the rails) from which the turnouts are built. The service life of rails, on the other hand, depends on many factors, such as their materials, operating characteristics, and constructional elements. When analysing the durability of a turnout in operation, it is necessary to consider the results of turnout observations. In the curves of a diverted track, the service life of the rails, depending on the radius of the curve, is much shorter. The ratio of the service life of rails laid in curves to the service life of rails on straight sections of track λ_s_ is determined by empirical formula (2) [8]:

(2) $$\lambda_{s}=-5.75\cdot 10^{-7}\cdot R^{2}+1.62\cdot R\cdot 10^{-3}-0.15$$

where R is the curve radius. The diagram presented in Figure 16 shows the effect of curves of the diverted tracks on the service life of rails considering the four most frequently used values of the diverted tracks’ radii at turnouts of 190, 300, 500, and 760 m.

This graph shows that turnouts featuring diverted tracks with a radius of 190 are the most vulnerable to degradation. Thus, for turnouts with a radius of 190 m, i.e., for Rkpd_iwew_ turnout No. 9, the value λ_190_ = 0.14. This value (λ_190_ = 0.14) for a turnout with a radius of 300 m, is twice as low at λ_300_ = 0.28 and more than five times as low for a radius of 760 m.

Several studies have been conducted in order to determine the relationship between turnouts and dynamic loads [7,8,11,13,48]. Hamarat et al. [48] presented new insights from multibody dynamic analyses of a turnout system under impact loads. A numerical model capable of determining impact forces was developed to evaluate the dynamic behaviors of a railway turnout and their effects on turnout components, such as bearers, ballasts, etc.

In [88], Kuźmiński et al. noted that the rapid increase in the world’s population and the interrelated increase in the number of vehicles means that the traffic on roads will continue to increase and the problem of traffic jams will become increasingly more critical. However, it should be noted that rail transport is a safer means of transport, characterized by properties that have a positive impact on increasing safety and environmental protection compared to road (car) transport.

This research showed an abnormality in the position of the geometric center SG of a double slip turnout, causing its deformation. The main reasons for the geometric irregularities of a turnout are as follows:

- The incorrect shape of the curvature of the turnout-diverted track and a change in its radius (these anomalies are reflected in the rate of wear of the turnout structural elements, especially the rails, changes in the state of the track gauge, and the cant parameters of the turnout track, switch sleeper wear, and peace-of-mind of driving);
- Incorrect maintenance of the track gauge and cant values, especially within a switch, with special attention given to the diverted tracks for running on a curve;
- The incorrect determination of the exploitation speed on the diverted and main tracks;
- The impact of centrifugal forces;
- The deregulation of spacing track axis values (including the intertrack space);
- The condition of the blades;
- An inadequately compacted ballast underneath the switch sleepers;
- The lack of straightness of the main tracks, under-tightened screws, and a bad state of fixings;
- The wear of the nose or wing rails and obtuse crossings;
- Irregularities in the track rails on the vertical and horizontal planes;
- Incorrectly conducted repair work during current maintenance, e.g., improving the track gauge parameters and replacing the switch sleepers;
- Incorrect assessment of the position of the geometric center of the turnout;
- Incorrect surveying of the turnout’s location;
- Incorrectly assembled turnout; and,
- The creep (rail movement) of rails before and after the turnout.

The presented SMDOST method fills in the gaps of the previously conducted monitoring of turnouts. This method contributes to improving safety as the most important component of the quality of railway infrastructure operation and it can facilitate the management of activities aimed at continuously improving safety. The present results of the developed SMDOST method with MMS provide support for the decision-making process in many areas of railway infrastructure measurements. The SMDOST method and the present results will have a significant impact on the Safety Management System. The basic elements of the SMDOST method with MMS entail an improvement of quality in terms of maintenance alongside safety improvements. These results are reflected in the components of SMS, in the present analysis and evaluation, and in the reduction of risk. The research results relate to the RAMS procedure for railways, a key issue of which is ensuring reliability and maintenance safety, as well as exploitation and maintenance quality.

## 5. Conclusions

Today’s available technologies to ensure safe railway infrastructure for the public make it necessary for owners, managers, contractors, and partners of infrastructure projects to monitor said infrastructure effectively. The main result of the present experimental research campaign showed a shift of 0.011 m in the exploitation position of the SG point in Rkpd_iwew_ compared to the actual state (during the total turnout exploitation). Based on the versine measurements, the difference between the existing R_i_ and the designed R_p_ radius was shown to be 35 m. The length of the main track also deviated from the actual state by a maximum value of 0.019 m. Additionally, a difference was found in the value of the main angle of the turnout α_p_ proper to its existing state, causing mowing–turning of the turnout at the SG point. This research also showed a tendency for the parameter of the track gauge to increase, especially on the diverted track–arched. This affected the changes in the position of the SG point of the double slip turnout.

The irregularities in turnout geometry and their damage were mainly due to a lack of symmetry, a large number of movable structural elements, and/or a lack of continuous support provided by an adequately dense ballast under turnout switch sleepers.

The method of surveying with reference to obtuse crossings based on geometric dependencies in the double and outside slip turnout was used for implementation and stocktaking work in the scope of running repairs on the turnout. The properly conducted surveying of slip turnouts will ensure their proper condition.

Deformations adversely affect exploitation work and change the position of the geometric center of the turnout in rail transport. The deformed geometrical systems of turnouts produce faster wear and tear of the track surface elements, turnouts, and rolling stock. This, in turn, causes the rolling stock to run violently, posing a threat to the safety of the train traffic. This research also showed the practicality and usefulness of this type of MMS measurement, especially in places that are difficult to access (e.g., obtuse crossings and check rails). The SMDOST method can significantly support owners, infrastructure managers, contractors, infrastructure project partners, and researchers in making decisions for more detailed analyses.

The conducted research is part of the surveying and diagnostic work in the discipline of civil engineering and rail transport.

## 6. Patents

Digital track gauge. Utility Model PL 067852 Y1 (Toromierz cyfrowy. Wzór użytkowy PL 067852 Y1).

Messages from the Patent Office RP, 6, (2015) Warsaw.

Wiadomości Urzędu Patentowego RP, 6, (2015) Warszawa.

Magnetic-measuring parallel motion protractor and its application. Patent PL 235051 B1. (Zespół przekładnic magnetyczno—pomiarowych do pomiaru parametrów geometrycznych torów i rozjazdów. Patent PL 235051 B1).

Messages from the Patent Office RP, 05, (2020) Warsaw.

Wiadomości Urzędu Patentowego RP, 05, (2020) Warszawa.

## Figures and Tables

**Table 1:** Results of the measurements of the Rkpd_iwew_ No. 9 ab/cd turnout type S49/1:9/190 while using the SMDOST method.

Name	Proper Condition	Existing Condition	Difference
Diverted track radius	R_p_ = 190 m	R_i_ ≈ 225 m	35 m
Horizontal versine of the diverted track ad	f_p_ = 0.726	f_1_ = f_iad_ = 0.612 m	0.114 m
Horizontal versine of the diverted track bc	f_p_ = 0.726	f_2_ = f_ibc_ = 0.612 m	0.114 m
Main angle of the turnout (between the main tracks axles 1′–SG–2′)	α_p_ = 7^g^04^c^46_6_^cc^	α_iab_ = 7^g^02^c^40_8_^cc^	0^g^02^c^05_8_^cc^
Main angle of the turnout (between the main tracks axles 3′–SG–4′)	α_p_ = 7^g^04^c^46_6_^cc^	α_icd_ = 7^g^06^c^19_0_^cc^	0^g^01^c^72_4_^cc^
Main track length 1′–3′ in the axis (in full)	|1′–3′|_p axis_ = 33.230 m	|1′–3′|_i axis_ = 33.249 m	0.019 m
Main track length 2′–4′ in the axis (in full)	|2′–4′|_p axis_ = 33.230 m	|2′–4′|_i axis_ = 33.226 m	0.004 m
Length of main track 1′–SG in axis	|1′–SG|_p axis_ = 16.615 m	|1′–SG|_i axis_ = 16.618 m	0.003 m
Length of main track SG–3′ in axis	|SG–3′|_p axis_ = 16.615 m	|SG–3′|_i axis_ = 16.631 m	0.016 m
Length of main track 2′–SG in axis	|2′–SG|_p axis_ = 16.615 m	|2′–SG|_i axis_ = 16.604 m	0.011 m
Length of main track SG–4′ in axis	|SG–4′|_p axis_ = 16.615 m	|SG–4′|_i axis_ = 16.622 m	0.007 m

**Table 2:** Values of the parameter track gauge and cant for the turnout pre-blade contact at points a and a_1_—periodic monitoring of Rkpd_iwew_ No 9 ab/cd type S49/1:9/190.

Turnout Number and Type Date of Measurement	Name of the Parameter	Turnout Side	Proper Condition and Permissible Deviation of the Parameter Track Gauge and Cant [mm]	
			Main Track	Main Track
			a	a_1_
	Track Gauge		$35_{-4}^{+8}$	$35_{-4}^{+8}$
	Track Cant		$0_{-12}^{+12}$	$0_{-12}^{+12}$
9 ab/cd S49/1:9/190 7 May 2018	Track gauge	ab	43	34
	Track gauge	cd	41	33
	Track cant	ab	−3	6
	Track cant	cd	0	−3
9 ab/cd S49/1:9/190 5 February 2019	Track gauge	ab	42	35
	Track gauge	cd	40	35
	Track cant	ab	−4	5
	Track cant	cd	1	−1
9 ab/cd S49/1:9/190 5 August 2019	Track gauge	ab	43	35
	Track gauge	cd	40	35
	Track cant	ab	2	−2
	Track cant	cd	0	−1
9 ab/cd S49/1:9/190 2 November 2019	Track gauge	ab	45	35
	Track gauge	cd	40	35
	Track cant	ab	−3	8
	Track cant	cd	1	2

The red color indicates the exceeded values.

**Table 3:** Values of the track gauge and cant parameter of the switch for points c, c_1_, c_2_, and c_3_—periodic monitoring of Rkpd_iwew_ No. 9 type S49/1:9/190.

Turnout Number and Type Date of Measurement	Name of the Parameter	Turnout Side	Proper Conditions and Permissible Deviation of the Parameter Track Gauge and Cant [mm]			
			Main Track	Diverted Track	Main Track	Diverted Track
			c	c_1_	c_2_	c_3_
	Track Gauge		$35_{-4}^{+8}$	$43_{-4}^{+14}$	$35_{-4}^{+8}$	$43_{-4}^{+14}$
	Track Cant		$0_{-12}^{+12}$	$0_{-12}^{+12}$	$0_{-12}^{+12}$	$0_{-12}^{+12}$
9 ab/cd S49/1:9/190 7 May 2018	Track gauge	ab	37	52	40	52
	Track gauge	cd	35	50	37	47
	Track cant	ab	−1	−1	−2	−3
	Track cant	cd	0	−1	0	0
9 ab/cd S49/1:9/190 5 February 2019	Track gauge	ab	38	52	40	52
	Track gauge	cd	35	50	37	44
	Track cant	ab	−1	−1	−3	−4
	Track cant	cd	−1	−1	−3	0
9 ab/cd S49/1:9/190 5 August 2019	Track gauge	ab	39	55	41	54
	Track gauge	cd	36	50	37	50
	Track cant	ab	0	0	0	1
	Track cant	cd	2	−3	2	−3
9 ab/cd S49/1:9/190 2 November 2019	Track gauge	ab	37	55	40	54
	Track gauge	cd	36	50	38	50
	Track cant	ab	0	1	−3	−3
	Track cant	cd	0	−1	0	0

**Table 4:** Values of the track gauge and cant parameters in the switch at points c, c_1_, c_2_, and c_3_—periodic monitoring of four additional Rkpd_iwew_ types S49/1:9/190.

Turnout Number and Type Date of Measurement	Name of the Parameter	Turnout Side	Proper Conditions and Permissible Deviation of the Parameter Track Gauge and Cant [mm]			
			Main Track	Diverted Track	Main Track	Diverted Track
			c	c_1_	c_2_	c_3_
	Track Gauge		$35_{-4}^{+8}$	$43_{-4}^{+14}$	$35_{-4}^{+8}$	$43_{-4}^{+14}$
	Track Cant		$0_{-12}^{+12}$	$0_{-12}^{+12}$	$0_{-12}^{+12}$	$0_{-12}^{+12}$
134 ab/cd S49/1:9/190 4 November 2019	Track gauge	ab	55	78	55	70
	Track gauge	cd	47	62	41	58
	Track cant	ab	1	4	4	8
	Track cant	cd	0	4	1	1
136 ab/cd S49/1:9/190 4 November 2019	Track gauge	ab	37	49	38	57
	Track gauge	cd	37	58	35	52
	Track cant	ab	−1	−2	0	1
	Track cant	cd	3	3	4	8
182 ab/cd S49/1:9/190 5 November 2019	Track gauge	ab	43	55	40	60
	Track gauge	cd	41	60	38	49
	Track cant	ab	15	12	11	15
	Track cant	cd	11	15	13	10
139 ab/cd S49/1:9/190 5 November 2019	Track gauge	ab	34	52	33	54
	Track gauge	cd	41	61	41	68
	Track cant	ab	0	0	0	0
	Track cant	cd	0	0	0	0

The red color indicates the exceeded values.
